# A Pathological Diagnosis Method for Fever of Unknown Origin Based on Multipath Hierarchical Classification: Model Design and Validation

**DOI:** 10.2196/58423

**Published:** 2024-12-09

**Authors:** Jianchao Du, Junyao Ding, Yuan Wu, Tianyan Chen, Jianqi Lian, Lei Shi, Yun Zhou

**Affiliations:** 1 School of Telecommunications Engineering, Xidian University, Xi'an, China; 2 Duke University Health System, Durham, NC, United States; 3 Department of Infectious Diseases, The First Affiliated Hospital of Xi’an Jiaotong University, Xi’an, China; 4 Department of Infectious Diseases, The Second Affiliated Hospital of Air Force Medical University, Xi'an, China

**Keywords:** fever of unknown origin, FUO, intelligent diagnosis, machine learning, hierarchical classification, feature selection, model design, validation, diagnostic, prediction model

## Abstract

**Background:**

Fever of unknown origin (FUO) is a significant challenge for the medical community due to its association with a wide range of diseases, the complexity of diagnosis, and the likelihood of misdiagnosis. Machine learning can extract valuable information from the extensive data of patient indicators, aiding doctors in diagnosing the underlying cause of FUO.

**Objective:**

The study aims to design a multipath hierarchical classification algorithm to diagnose FUO due to the hierarchical structure of the etiology of FUO. In addition, to improve the diagnostic performance of the model, a mechanism for feature selection is added to the model.

**Methods:**

The case data of patients with FUO admitted to the First Affiliated Hospital of Xi’an Jiaotong University between 2011 and 2020 in China were used as the dataset for model training and validation. The hierarchical structure tree was then characterized according to etiology. The structure included 3 layers, with the top layer representing the FUO, the middle layer dividing the FUO into 5 categories of etiology (bacterial infection, viral infection, other infection, autoimmune diseases, and other noninfection), and the last layer further refining them to 16 etiologies. Finally, ablation experiments were set to determine the optimal structure of the proposed method, and comparison experiments were to verify the diagnostic performance.

**Results:**

According to ablation experiments, the model achieved the best performance with an accuracy of 76.08% when the number of middle paths was 3%, and 25% of the features were selected. According to comparison experiments, the proposed model outperformed the comparison methods, both from the perspective of feature selection methods and hierarchical classification methods. Specifically, brucellosis had an accuracy of 100%, and liver abscess, viral infection, and lymphoma all had an accuracy of more than 80%.

**Conclusions:**

In this study, a novel multipath feature selection and hierarchical classification model was designed for the diagnosis of FUO and was adequately evaluated quantitatively. Despite some limitations, this model enriches the exploration of FUO in machine learning and assists physicians in their work.

## Introduction

### Background

Fever of unknown origin (FUO) is a medical term used to describe a group of diseases that exhibit a prolonged fever lasting for a duration exceeding 3 weeks and that cannot be diagnosed even after 1 week of outpatient or inpatient examinations. This concept was initially introduced by Petersdorf and Beeson [1]. The etiology of FUO is multifactorial and encompasses a wide range of factors, including over 200 different species [2], such as *Streptococcus pneumoniae* [3], peritoneal mesothelioma [4], and *Bacteroides fragilis* [5]. The distribution of these causative agents varies temporally and geographically, necessitating comprehensive and in-depth investigations to determine the underlying cause of the disease accurately. Consequently, identifying the cause of FUO poses a significant challenge within the medical field [6]. In diagnosing febrile illness, doctors must conduct a thorough evaluation and examination based on the patient’s symptoms, signs, and possible causes to determine the final diagnosis and treatment plan. However, despite conducting a comprehensive examination, it has been found that one-third of patients presenting with persistent fever remain undiagnosed [7].

With the continuous progress of machine learning (ML), its application in various domains of production and business activities has experienced substantial growth [8-10]. In the realm of medicine, the use of ML-based disease diagnosis technology holds immense importance, as it aids in enhancing the accuracy and real-time capabilities of doctors’ diagnoses. In recent years, there has been a significant increase in the advancement of intelligent diagnostic techniques that use ML algorithms to independently predict potential causes of diseases. Choudhury [11] uses a logistic regression (LR) model to diagnose cases of malignant pleural mesothelioma. Ogunleye and Wang [12] propose a liver disease classification approach that uses the extreme gradient boosting and further improves its performance by optimizing its parameters using a genetic algorithm. It can be seen that intelligent diagnosis has received significant attention in recent years. More researches are illustrated in Table 1, including medical image analysis [13-21], pathology analysis [19,21-24], and diagnostic system [25-30].

**Table 1.** The review of intelligent diagnostic methods in health care.

Diseases	Dataset type	Method	Study
Parkinson	Tabular	Random forest	Polat [25]
COVID-19	X-ray image	Ensemble algorithms	Sunnetci and Alkan [13]
Lung cancer	CT^a^ image	ISO^b^-CNN^c^	Yan and Razmjooy [14]
Epilepsy	EEG^d^ signals	CNN-RNN^e^	Malekzadeh et al [26]
COVID-19	X-ray image	Fusion of CNN, SVM^f^, and Sobel filter	Sharifrazi et al [15]
COVID-19	X-ray image	UA-ConvNet^g^	Gour and Jain [16]
COVID-19	CT image	CycleGan^h^	Ghassemi et al [17]
COVID-19	CT image	CNN and transfer learning	Balaha et al [18]
Breast cancer	Tabular	(SVM + LR^i^ + NB^j^ + DT^k^) +ANN^l^	Naseem et al [22]
Lung cancer	Sequences	MGGP^m^	Sattar et al [23]
Gastric cancer	Endoscopic images	GAIN-ResNet-50^n^, CA-U-Net^o^	Ma et al [24]
Lung cancer	PET^p^ or CT image	Logistic regression	Wang et al [19]
Lymph node metastasis	Ultrasound images	YOLO^q^	Fu et al [20]
Breast cancer	Histopathology images	VGG^r^	Yuan et al [21]
Schizophrenia	EEG signals	CNN-LSTM^s^	Shoeibi et al [27]
Schizophrenia and attention-deficit/hyperactivity disorder	rs-fMRI^t^	Convolutional autoencoder-interval and type-2 fuzzy regression	Shoeibi et al [28]
Autism spectrum disorder	EEG and eye-tracking signals	Machine learning	Wadhera [29]
Epilepsy	EEG signals	CNN-LSTM	Wang et al [30]

a CT: computed tomography.

b ISO: improved snake optimization.

c CNN: convolutional neural network.

d EEG: electroencephalography.

e RNN: recurrent neural network.

f SVM: support vector machine.

g UA-ConvNet: uncertainty-aware convolutional neural network.

h CycleGan: cycle-consistent generative adversarial network.

i LR: logistic regression.

j NB: naive Bayes.

k DT: decision tree.

l ANN: artificial neural network.

m MGGP: multigene genetic programming.

n GAIN-ResNet-50: guided attention inference network-residual network-50.

o CA-U-Net: convolutional block attention module and atrous spatial pyramid pooling module based on U-Net.

p PET: positron emission tomography.

q YOLO: you only look once.

r VGG: visual geometry group.

s LSTM: long short-term memory.

t rs-fMRI: resting-state functional magnetic resonance imaging.

### Objectives

Due to the current major applications being primarily binary classification or a few class classification problems, algorithm designs are often directly aimed at all classes using flat classification methods. However, FUO can be attributed to numerous potential causes, necessitating a multiclass classification approach. Consequently, using flat classification methods alone results in suboptimal accuracy and fails to fulfill the application’s requirements [31]. Given the hierarchical structure of the etiological labels in the FUO dataset, it is possible to use hierarchical classification [32] models to analyze the dataset. By leveraging the hierarchical associations among data classes, a top-down methodology is used for hierarchical classification, culminating in acquiring the corresponding class at the leaf level. Hierarchical classification involves decomposing a multiclass task into several subclassification tasks, resulting in a simplified model and reduced complexity in modeling. Additionally, it presents a notable advantage in terms of computational efficiency for both classification learning and prediction tasks. This characteristic makes it particularly suitable for fulfilling the requirements of etiological prediction in cases of FUO.

Therefore, we introduce a novel auxiliary diagnostic method for FUO using multipath feature selection and hierarchical classification. The data will be organized into a hierarchical structure based on disease classes for hierarchical classification. Subsequently, prediction will be conducted from the highest to the lowest level until the final classification class is achieved. To mitigate the likelihood of ineffective lower-level classification resulting from errors in higher-level classification, the hierarchical classification process incorporates multiple path prediction models with controllable preselected classes. This approach aims to enhance the accuracy of lower-level classification. Additionally, the L_1,2_ regularization constraint [33] is used for feature selection at each level to eliminate redundant features and minimize interference, thereby enhancing prediction accuracy.

## Methods

### Framework

#### Overview

**Figure 1.**
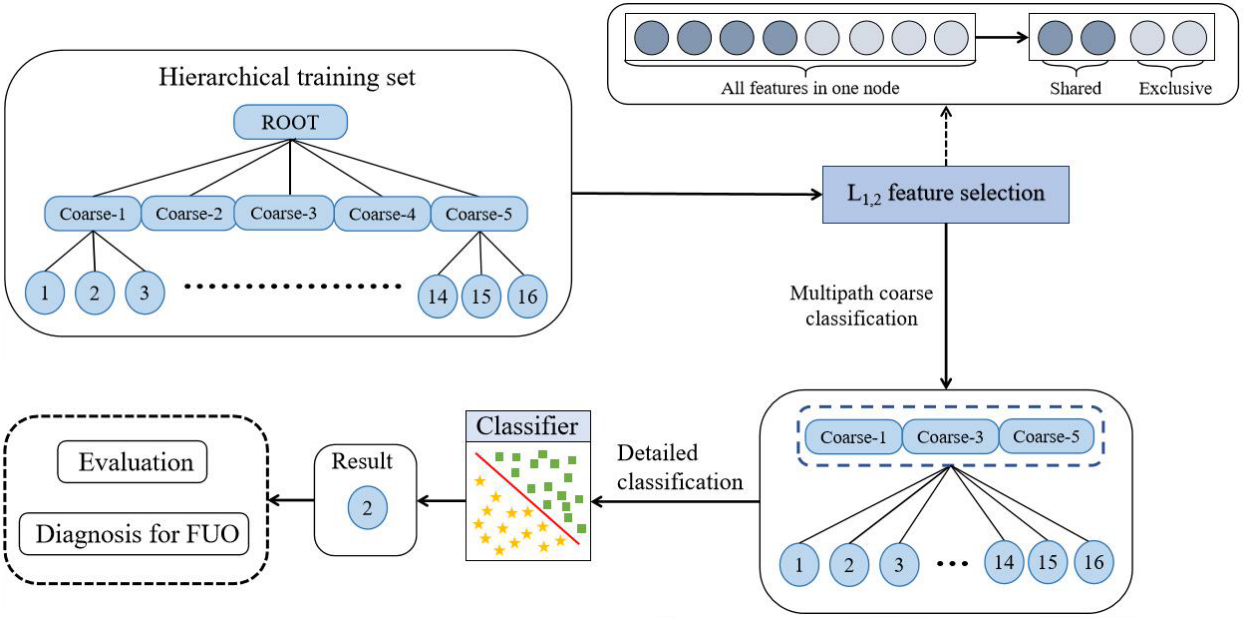
The framework of our method. FUO: fever of unknown origin.

The framework of the hierarchical classification method based on multipath and feature selection proposed in this paper is illustrated in Figure 1. The process can be divided into two steps: (1) feature selection is performed at each layer using L_1,2_ regularization constraints based on the tree hierarchy to eliminate redundant features and reduce interference and (2) hierarchical classification is then performed using the selected features, and multipath prediction models are built by preselecting controllable multiple classes during the hierarchical classification process.

For a more detailed explanation of the multipath hierarchical classification process, please refer to Multimedia Appendix 1 [34].

#### Hierarchical Feature Selection

Traditional feature selection assumes that all classes are independent of each other, and a set of standard features is selected for all classes to form a subset of features before classification. However, Freeman et al [35] found that certain features are more suitable for classifying some classes with better discriminative properties. On the other hand, these features do not improve the classification performance for other classes. Feature selection in hierarchical structures allows for selecting a distinct subset of features for each subcategorization task within the structure. This approach enhances the performance of the classification task.

We select an L_1,2_ regularization constraint for each level of the tree hierarchy, and feature ranking is performed to select the most relevant features. L_1,2_ regularization constraint is an unbiased estimation that results in a sparser and more computationally efficient solution to the minimization problem than L_1_ regularization [33].

In addition, traditional hierarchical feature selection considers different nodes as independent. It selects completely different subsets of features, whereas, in this paper, we propose selecting a portion of the same feature for every layer, known as shared features identified by *W_i_*, for different nodes in the same layer. Additionally, we select exclusive features for each node that are suitable for classification identified by *D_i_*. These 2 matrices are solved by the optimization method.

#### Multipath Hierarchical Classification

The framework shows that after the hierarchical feature selection based on L_1,2_ paradigm regularization, these features are used as the feature subset for classification. Subsequently, *k* candidate coarse classifications are selected from the coarse classifications (in Figure 1, *k*=3) by probabilities from LR. The candidate coarse classifications are the top *k* most likely to be the broad category of etiology to which the disease belongs (eg, bacterial infection and viral infection). The etiologies to be identified are targeted to specific categories under these coarse classifications. Refined categorization follows, leading to the final diagnosis.

### Dataset

#### Information

The dataset used in our research is obtained from the clinical diagnostic records of patients with FUO admitted to the First Affiliated Hospital of Xi’an Jiaotong University between 2011 and 2020 in China. Each sample in this study represents authentic clinical data obtained from patients with FUO, encompassing pathological data and diagnoses provided by physicians. The pathological data encompass a range of information, including clinical symptoms, epidemiological history, past medical history, laboratory tests, medical imaging, and indicators from pathological examination. The statistical indicators of the dataset are presented in Table 2.

For this study, we used patients’ pathological data and doctors’ diagnostic results as the training dataset to construct the model. Due to the limited quantity of available data, there is a possibility of encountering a significant imbalance within the dataset. This imbalance may result in a bias toward predicting classes with more extensive data samples, ultimately impacting the overall classification performance. During the data analysis process, samples that contained less than 6 instances of a particular disease were excluded to address the imbalance issue. After the refinement process, a final dataset of 564 samples was obtained. This dataset encompasses 5 coarse etiologies (bacterial infection, viral infection, other infection, autoimmune diseases, and other noninfection), and 16 exact etiologies belong to them. Please consult Table 3 for more detailed information regarding the dataset.

**Table 2.** The statistical analysis of the data (collected during 2011‐2020).

Indicators		Samples, n (%)
**Sex**		
	Male	303 (53.7)
	Female	261 (46.3)
**Age (years)**		
	0-20	87 (15.4)
	20-40	172 (30.5)
	40-60	188 (33.3)
	>60	117 (20.8)
Infection		399 (70.7)
Noninfection		165 (29.3)

**Table 3.** The breakdown of etiologies included in the dataset.

Diagnose	Values, n (%)	Label
**Bacterial infection**		
Liver abscess	24 (4.3)	1
Endocarditis	12 (2.1)	2
Brucellosis	64 (11.4)	3
**Viral infection**		
Epstein-Barr virus infection	77 (13.7)	4
Cytomegalovirus infection	14 (2.5)	5
Infectious mononucleosis	38 (6.7)	6
Other viral infection	103 (18.3)	7
**Other infection**		
Kala-azar	11 (1.9)	8
Mycoplasma infection	11 (1.9)	9
Rickettsia infection	45 (8)	10
**Autoimmune diseases**		
Anca-associated vasculitis	12 (2.1)	11
Adult-onset Still disease	20 (3.5)	12
Lymphoma	33 (5.9)	13
**Other noninfection**		
Systemic inflammatory response syndrome	47 (8.3)	14
Hemophagocytic syndrome	19 (3.4)	15
Necrotizing lymphadenitis	34 (6)	16

#### Hierarchy Label

**Figure 2.**
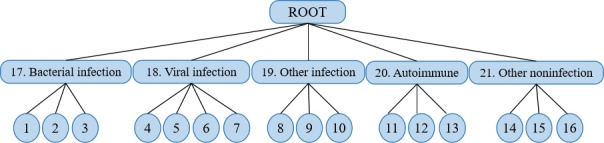
The hierarchical structure of classes.

According to the pathological characteristics of FUO, the dataset can be organized in a hierarchical structure tree [36]. The categories of the FUO tree span from abstract etiology to concrete etiology, progressing from the root node to the leaf nodes in a top-to-bottom manner. The hierarchical tree structure in the dataset exhibits a 3-tiered system of granularity. The first layer, “ROOT,” signifies FUO, while the subsequent layer categorizes FUO into 5 classifications: bacterial infection, viral infection, other infection, autoimmune diseases, and other noninfections, labeled from 17 to 21. The final layer further delineates these 5 categories into specific etiologies. For instance, within the bacterial infection labeled as 17, liver abscess, endocarditis, and brucellosis are identified and assigned labels 1‐3, respectively, as illustrated in Table 3. This process is similarly applied to the other 4 middle categories, culminating in the hierarchical structure tree presented in Figure 2.

#### Data Preprocessing

The clinical symptoms, epidemiological history, past medical history, and laboratory tests in the pathological data of the patients with FUO exhibited a range of different forms, including continuous and discrete data. Therefore, the data underwent preprocessing to ensure standardization.

##### Imputation of Missing Values

The k-nearest neighbor was used to interpolate continuous missing data to address missing values in the dataset [37]. For discrete missing data, we used the mode of all available data points within the respective data item.

##### Coding

After filling in the missing values in the discrete data, it was necessary to identify the category features that lack significance in size. Subsequently, numerical or vectorization operations can be applied to these features. Two coding methods, namely 0‐1 encoding and one-hot encoding, were used in the paper.

##### Normalization

A process applied to continuity data after filling in missing values, aiming to distribute the values on the [0,1] interval to result in the preprocessed normalized data [38]:


(1) $$x^{\prime }=\frac{x-MIN}{MAX-MIN}$$

where *x* represents the original continuity data, MIN represents the minimum value of the data item within its respective location, and MAX represents the maximum value of the data item within its respective location. After undergoing data preprocessing, the final dataset consists of 564 samples in total, with each sample having 327 dimensions.

### Experimental Settings

We input body indicators after data preprocess as features into the model to get the etiological diagnosis. To fully evaluate our method, we designed 2 types of experiments: ablation experiments and comparison experiments. Ablation experiments were to determine the optimal structure of our method, and comparison experiments were to compare the advantages and disadvantages of our method with other methods.

Ablation experiments consisted of 2 experiments. Experiment 1 selected support vector machine (SVM) and random forest (RF) as classifiers and evaluated the effect of the number of hierarchical paths, with the optional number ranging from 1 to 5. Based on the optimal number of hierarchical paths from experiment 1, experiment 2 evaluated the effect of the different feature selection ratios of the L_1,2_ regularization using SVM. We set the ratio starting from 0.05 and ending at 1 (with an interval of 0.05). After the ablation experiments to determine the optimal number of hierarchical paths and the ratio of feature selection to determine the optimal structure, it turned to comparison experiments.

Three experiments were set up for the comparison experiments. Experiment 3 compared the performance of L_1,2_ regularization proposed in this paper with 10 feature selection methods, and the evaluation metric was the accuracy. Experiment 4 compared our method with 7 ML models and 4 hierarchical classification algorithms. The comparison metric with ML models was accuracy, and with hierarchical classification algorithms was not only accuracy but also hierarchical *F*_1_-measure (*F*_H_), *F*_H_ based on the closest ancestor (*F*_LCA_), and tree-induced error (TIE). In addition, we also refined the comparison to 16 etiologies involving 4 hierarchical classification algorithms with the metric of accuracy. Experiment 5 compared our method with the hierarchical classification methods on 5 broad etiologies, again with the metric of accuracy.

### Compared Methods

To evaluate the performance of our method, experiments were conducted and compared with 4 similar hierarchical classification methods:

1. Top-down LR hierarchical classification: At each granularity level, the node with the highest predicted probability is selected as the classification result, recursively performing from top to bottom until reaching the leaf level.
2. Hierarchical classification based on optimal N-paths (HNBP) [39]: The proposed approach converts the task of class prediction into a search problem, aiming to identify multiple paths within a tree-like hierarchy with the highest joint probability. This strategy effectively mitigates the issue of error propagation between different levels.
3. Cost-sensitive hierarchical classification based on class hierarchy correlation [40]: In the same layer of hierarchical classification, there is an imbalanced data distribution, introducing cost-sensitive factors to reduce the tendency of majority class classification and improving the classification accuracy of minority classes.
4. Cost-sensitive hierarchical classification based on multiscale information entropy [41]: The computation of information entropy for various classes at each level of the hierarchy is performed, and an entropy threshold is established to mitigate the propagation of errors from higher-level classification tasks to lower-level ones. It assigns different cost weights to classes based on hierarchical information entropy to address data imbalance.

### Evaluation Metrics

The performance of the proposed method was assessed and confirmed through a series of experiments. Five metrics were used for evaluation: *F*_H_ [42], *F*_LCA_ [43], TIE [44], accuracy, and runtime (*T*). For details on calculating the indicators, please refer to Multimedia Appendix 2.

### Ethical Considerations

This study was approved by the institutional review board of Tangdu Hospital, Air Force Medical University (TDLL-202411-02). The study was conducted in accordance with the Declaration of Helsinki, and all participants gave their informed consent for inclusion before they participated in the study. Meanwhile, the data used in our study were deidentified to protect the privacy and confidentiality of the participants. This study did not provide compensation to the participants.

## Results

### Ablation Experiments

#### The Validation of the Hierarchical Paths

Our study compared the performance of the intermediate paths on the effectiveness of exact etiology categorization. The intermediate paths represent the process in the hierarchical structure from the root node through the second level of the coarse etiologies and finally to the specific etiologies. Within the context of the hierarchical classification method proposed in this paper, we selected the whole feature to assess the performance of intermediate path numbers while maintaining consistency. For the base classifiers, we chose SVM and RF. Empirical findings are presented in Table 4.

From the results of our method, the case of *k*=1 in Table 4 is equivalent to using the traditional single-path hierarchical classification method. In this case, the accuracy, *F*_H_, *F*_LCA_, and TIE metrics using SVM are 66.66%, 82.03%, 79.90%, and 60.8, respectively. The performance is the lowest among the results for different numbers of paths, as evidenced by the highest TIE. However, the *T* of 0.87 seconds is the shortest for this case, thanks to the single-path hierarchical approach that simplifies the model. When *k*=5, this scenario is equivalent to directly flattening the dataset for classification, as this paper only has 5 coarse categories. The accuracy of our method by SVM is 68.47%, which aligns closely with the SVM outcomes of various classification algorithms shown in Table 5, thus validating the earlier inference. Although, in this case, the accuracy of our method by RF is 13.48% higher than that of Table 5, it is due to the random nature of the classification mechanism of RF.

In contrast, the optimal hyperplane sought by SVM is constrained by the spatial distribution of the samples. Consequently, the outcomes of each search are relatively similar. Therefore, this discrepancy does not impact the conclusion that it is comparable to the direct flat classification of the dataset in the previous instance *k*=5. By comparing the classification results of multiple paths, it can be observed that both our method by SVM and RF exhibit the best performance when *k*=3. The accuracy of our method by SVM is 72.35%, representing an improvement of 5.69% and 3.88% compared to the single-path hierarchical classification with *k*=1 and the similar flattened classification with *k*=5, respectively. The accuracy of our method by RF is 69.20%, showing an improvement of 2.54% and 4.12% over the 2 approaches mentioned earlier. The results of both classifiers demonstrate that the multipath hierarchical classification approach can reduce the interlayer error propagation problem.

Additionally, decomposing the total task into multiple subtasks can reduce the complexity of the problem and improve the classification results. The running times of SVM and RF are 4.17 and 45.19 seconds, respectively. These times are 3.3 and 41.88 seconds more extended than the single-path hierarchical classification, suggesting that more paths will increase the hierarchical model’s complexity, prolonging the system’s decision time. However, it is still within an acceptable range.

**Table 4.** The performance comparison of intermediate path numbers on 16 detailed etiologies.

Classifier and path	Accuracy (%)	*F*_H_^a^ (%)	*F*_LCA_^b^ (%)	TIE^c^	*T* (seconds)
**Support vector machine**					
1	66.66	82.03	79.90	60.8	0.87
2	71.49	84.49	82.74	52.6	2.18
3	72.35	85.01	83.29	50.8	4.17
4	71.83	84.48	82.85	52.6	7.84
5	68.47	82.77	80.87	58.4	10.00
**Random forest**					
1	66.66	82.03	79.90	60.8	3.31
2	68.97	83.05	81.18	57.4	43.20
3	69.20	83.25	81.36	56.8	45.19
4	64.90	80.45	78.53	66.2	50.63
5	65.08	80.15	78.44	67.2	50.94

a *F*_H_: hierarchical *F*_1_-measure.

b *F*_LCA_: *F*_H_ based on the closest ancestor.

c TIE: tree-induced error.

**Table 5.** The accuracy of different classification methods on 16 detailed etiologies.

Method	Accuracy (%)
LR^a^	69.86
SVM^b^	68.46
KNN^c^	51.38
RF^d^	51.6
DT^e^	53.36
XGB^f^	62.22
ELM^g^	70.69
TDLR^h^	66.67
CSHCIC^i^	68.93
CSHC^j^	67.87
HNBP^k^	70.45
Our method	76.08

a LR: logistic regression.

b SVM: support vector machine.

c KNN: k-nearest neighbor.

d RF: random forest.

e DT: decision tree.

f XGB: extreme gradient boosting.

g ELM: extreme learning machine.

h TDLR: top-down logistic regression hierarchical classification.

i CSHCIC: cost-sensitive hierarchical classification based on class hierarchy correlation.

j CSHC: cost-sensitive hierarchical classification based on multiscale information entropy.

k HNBP: hierarchical classification based on optimal N-paths.

#### The Validation of Different Feature Selection Percentages

A comparison was conducted to evaluate the performance of various feature selection percentages. The selection of features at each level of the hierarchical tree structure was consistent, with an equal percentage being chosen.

**Figure 3.**
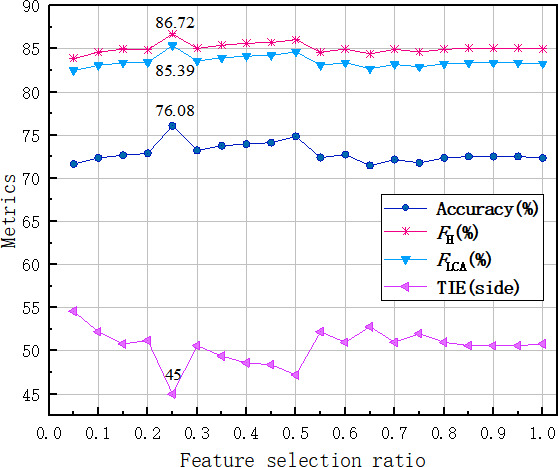
The performance comparison of different percentage feature selections on 16 detailed etiologies. *F*_H_: hierarchical *F*_1_-measure; *F*_LCA_: *F*_H_ based on the closest ancestor; TIE: tree-induced error.

From Figure 3, when the feature selection reaches 25%, the highest level of performance is attained, with an accuracy of 76.08%, *F*_H_ of 86.72%, *F*_LCA_ of 85.39%, and TIE reduced to 45. When the ratio ranges from 5% to 25%, accuracy, *F*_H_, and *F*_LCA_ show an increasing trend, while TIE shows a decreasing trend. However, when the percentage exceeds 25%, the trend of the 4 metrics reverses because selecting too many features may lead to overfitting and increased computational complexity. However, choosing too few features may result in underfitting and information loss. The optimal number of features balances model complexity and information retention, enhancing model generalization and performance.

### Comparison Experiments

#### The Validation of Different Feature Selection Methods

A comparison test of feature selection methods was performed to demonstrate the effectiveness of L_1,2_ feature selection. The selected comparison methods included traditional methods [45] like Fisher score and recursive feature elimination, as well as swarm intelligence methods [46-50] including whale optimization algorithm, particle swarm optimization, rat swarm optimization, Lévy flight trajectory–based whale optimization algorithm, improved discrete laying chicken algorithm, fuzzy entropy [51], L_1_ regularization, and L_2_ regularization. As shown in Table 6, L_1,2_ regularization achieves the highest accuracy of 72.14% and filters out approximately 30% of the features.

**Table 6.** The accuracy and number of features of different feature selection methods on 16 detailed etiologies.

Method	Values, n (%)	Accuracy (%)
FS^a^	326 (97.9)	64.02
RFE^b^	243 (73)	65.96
L_1_	35 (10.5)	60.84
L_2_	94 (28.2)	67.92
FE^c^	224 (67.3)	65.25
WOA^d^	145 (43.5)	68.64
PSO^e^	162 (48.6)	71.17
RSO^f^	135 (40.5)	68.19
LWOA^g^	258 (77.5)	71.28
IDLCA^h^	196 (58.9)	68.26
L_1,2_	229 (68.8)	72.14

a FS: Fisher score.

b RFE: recursive feature elimination.

c FE: fuzzy entropy.

d WOA: whale optimization algorithm.

e PSO: particle swarm optimization.

f RSO: rat swarm optimization.

g LWOA: Lévy flight trajectory–based whale optimization algorithm.

h IDLCA: improved discrete laying chicken algorithm.

#### The Validation of the Classification Methods

A comparison was conducted to assess the performance differences between the proposed method and alternative approaches. The proposed method used a value of 3 for the number of paths (*k*), used SVM as the base classifier, and set the feature selection percentage to 25%. To evaluate the accuracy of the assessment, a variety of comparison methods were used, including both hierarchical classification techniques and flat classification techniques such as LR, k-nearest neighbor, RF, SVM, extreme gradient boosting [52], and extreme learning machine (ELM) [53], as depicted in Table 5.

The results indicate that LR, SVM, and ELM demonstrated relatively high performance compared to other flat classification methods. In contrast, the remaining flat methods exhibited lower accuracy due to their ability to prevent overfitting. LR and SVM improve generalization by regularizing and maximizing classification intervals, while the kernel method of SVM allows capturing nonlinear features in high-dimensional space. ELM prevents the model from falling into local optima by random initialization and fast training and combines linear and nonlinear properties to achieve effective feature selection. Overall, these models can flexibly handle complex relationships in high-dimensional data and perform well in the high-dimensional classification of small-sample data.

On the contrary, the hierarchical classification methods demonstrated strong performance, with the proposed method exhibiting the highest level of effectiveness, surpassing all other alternative approaches.

About the *F*_H_ and *F*_LCA_ metrics, the proposed method was compared to other hierarchical classification methods, and the outcomes are depicted in Table 7. The results indicate that our method achieved the highest rankings in both metrics. It obtained an *F*_H_ of 86.72%, 2.63% higher than the second-ranked HNBP, and achieved an *F*_LCA_ of 85.39%, surpassing HNBP by 3.2%. Our method demonstrated the lowest TIE with a value of 45, representing a significant decrease of 8.6 compared to the second-ranked HNBP. This observation shows that our approach exhibits fewer misclassifications and superior classification performance.

**Figure 4.**
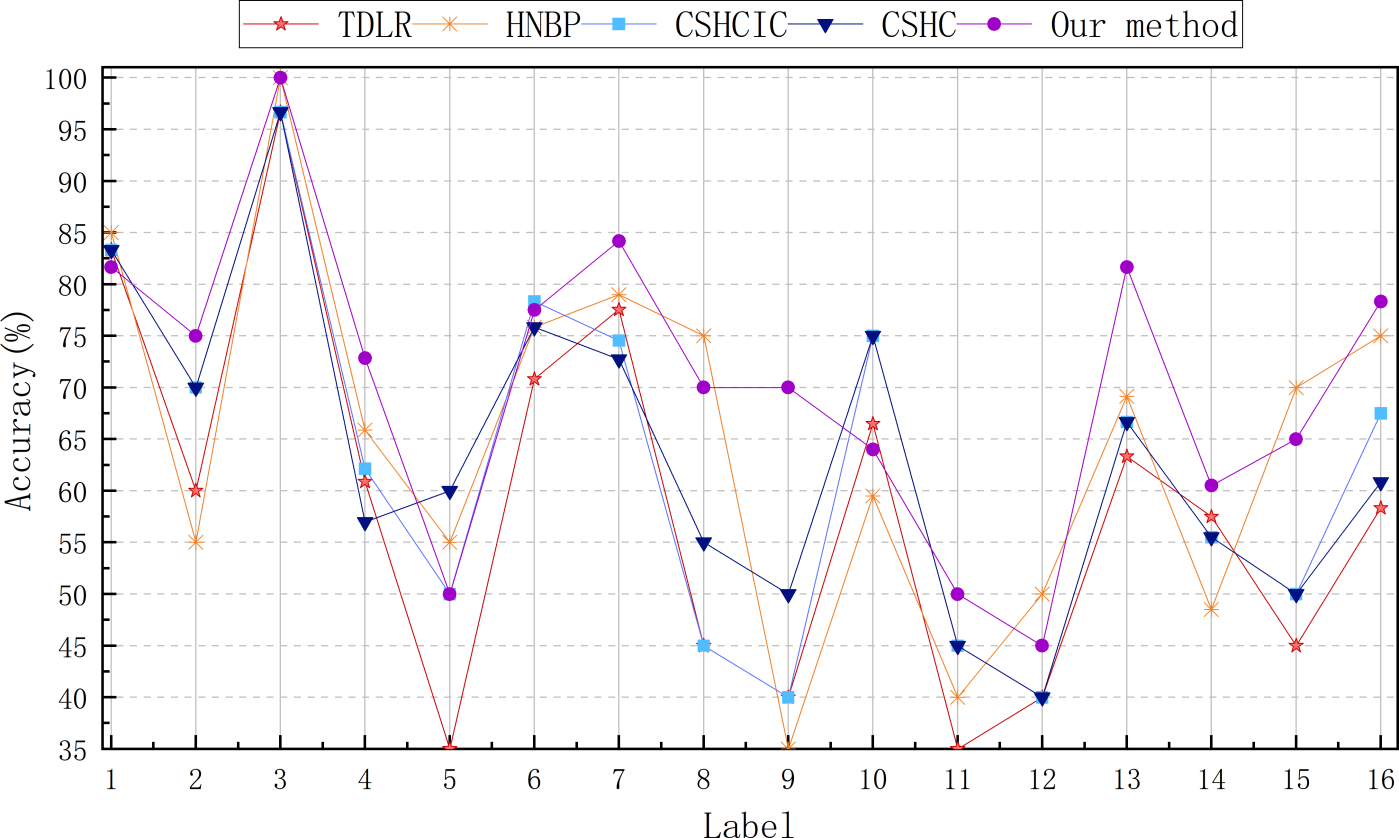
The accuracy of different hierarchical methods on 16 detailed etiologies respectively. CSHC: cost-sensitive hierarchical classification based on multiscale information entropy; CSHCIC: cost-sensitive hierarchical classification based on class hierarchy correlation; HNBP: hierarchical classification based on optimal N-paths; TDLR: top-down logistic regression hierarchical classification.

Figure 4 compares our method’s classification outcomes with other hierarchical classification algorithms to evaluate the accuracy of different classes. Based on the obtained results, it is evident that our method demonstrates superior classification accuracy across the majority of classes.

**Table 7.** The validation of different hierarchical methods on 16 detailed etiologies.

Method	*F*_H_^a^ (%)	*F*_LCA_^b^ (%)	TIE^c^
TDLR^d^	82.03	79.9	60.8
CSHCIC^e^	83.08	81.18	57.2
CSHC^f^	82.25	80.42	60
HNBP^g^	84.09	82.19	53.6
Our method	86.72	85.39	45

a *F*_H_: hierarchical *F*_1_-measure.

b *F*_LCA_: *F*_H_ based on the closest ancestor.

c TIE: tree-induced error.

d TDLR: top-down logistic regression hierarchical classification.

e CSHCIC: cost-sensitive hierarchical classification based on class hierarchy correlation.

f CSHC: cost-sensitive hierarchical classification based on multiscale information entropy.

g HNBP: hierarchical classification based on optimal N-paths.

Additionally, we compared the accuracy of the coarse classes at the intermediate level of the FUO dataset, as shown in Table 8. Our method outperforms other hierarchical classification algorithms regarding accuracy across all 5 coarse classes. The observation above suggests that our method’s implementation successfully addresses the error propagation issue. In the context of class 17, our method demonstrates a prediction accuracy of approximately 98%. In class 20, our method shows the most significant improvement compared to other methods, exhibiting a 15% improvement over the top-down LR hierarchical classification and an almost 8% improvement over the HNBP. Despite the relatively low overall prediction accuracy observed in all methods for classes 19‐21, our method demonstrates a notable improvement in the prediction accuracy of these 3 classes, surpassing 70%. This finding suggests that the proposed algorithm significantly enhances the predictive performance. From the analysis of the accuracy metrics, it is apparent that misclassified test samples exist. This can be attributed to several factors, including the imbalanced distribution of samples across different classes in the dataset, the inherent variability in the sample data, and the inadequate cleaning resulting in sample overlap.

**Table 8.** The accuracy of different hierarchical methods on 5 coarse etiologies (%).

Label	TDLR^a^ (%)	HNBP^b^ (%)	CSHCIC^c^ (%)	CSHC^d^ (%)	Our method (%)
17	96.73	94.55	94.61	93.82	97.96
18	87.05	88.95	89.50	89.38	89.72
19	66.67	65.03	67.06	65.01	71.18
20	61.57	68.86	62.90	60.17	76.75
21	68.28	71.68	68.53	68.52	74.75
Mean (SD)	76.06 (15.06)	77.81 (13.09)	76.52 (14.44)	75.38 (15.18)	82.07 (11.31)

a TDLR: top-down logistic regression hierarchical classification.

b HNBP: hierarchical classification based on optimal N-paths.

c CSHCIC: cost-sensitive hierarchical classification based on class hierarchy correlation.

d CSHC: cost-sensitive hierarchical classification based on multiscale information entropy.

To facilitate a comprehensive examination of the pivotal “multipath prediction” aspect of our method, Table 9 provides the progress of 20 samples from the middle to final layers to the final prediction versus the ground truth. The analysis reveals that 14 samples were predicted correctly, including liver abscess, brucellosis, viral infection, rickettsia infection, lymphoma, and necrotizing lymphadenitis (corresponding to labels 1, 3, 7, 10, 13, and 16). Additionally, Epstein-Barr virus infection and systemic inflammatory response syndrome (corresponding to labels 4 and 14) were predicted with partial accuracy. Among the 6 samples that were incorrectly predicted, samples 11, 17, and 19 were classified within the same broad disease category as their corresponding true values. For example, sample 11 was predicted as label 14. At the same time, its ground truth was label 16, both of which fall under middle layer label 21, the category with the highest likelihood ranking among the 3 nodes in the middle layer.

**Table 9.** The visualization of the multipath prediction process from the middle to the last layers, then to the final diagnostic results.

ID	Middle layer	Last layer, label (possible [%])	Prediction	Ground truth
1	18, 21, 19	7 (93.29), 4 (2.79), 16 (2.06)	7	7
2	21, 19, 18	16 (84.69), 10 (6.62), 14 (4.27)	16	16
3	18, 20, 21	7 (77.33), 13 (10.38), 14 (4.31)	7	7
4	17, 18, 19	1 (59.27), 3 (14.31), 4 (10.98)	1	1
5	18, 21, 19	7 (95.51), 4 (1.64), 5 (1.42)	7	7
6	18, 21, 17	7 (91.73), 4 (3.30), 15 (1.71)	7	7
7	19, 21, 18	10 (88.07), 8 (4.54), 14 (3.34)	10	10
8	18, 20, 21	4 (66.27), 6 (23.41), 7 (3.57)	4	4
9	20, 18, 21	13 (72.37), 11 (9.47), 6 (6.80)	13	13
10	17, 18, 21	3 (83.08), 1 (7.28), 4 (5.92)	3	3
11	21, 19, 20	14 (60.37), 16 (26.64), 10 (7.35)	14	16
12	18,19,21	7 (50.20), 10 (38.56), 5 (7.64)	10	10
13	21,19,20	14 (58.29), 10 (27.01), 16 (9.03)	14	12
14	18,19,20	7 (35.15), 6 (28.92), 10 (20.66)	10	10
15	18,19,21	7 (50.20), 10 (38.56), 5 (7.64)	10	10
16	21, 19, 18	15 (40.78), 14 (25.63), 8 (17.48)	8	8
17	18, 21, 20	6 (79.28), 4 (8.03), 5 (5.09)	4	6
18	21, 18, 19	14 (79.74), 15 (9.76), 9 (3.24)	14	9
19	18, 21, 17	4 (70.70), 15 (10.90), 5 (9.80)	4	7
20	19, 21, 18	10 (36.13), 14 (28.45), 12 (15.62)	14	12

## Discussion

### Principal Findings

This paper proposes a hierarchical classification method based on multipath and feature selection for intelligent diagnosis of FUO. The method innovatively introduces the L_1,2_ constraint feature selection method and extends the single path of the hierarchical classification method to incorporate multiple paths. Our method achieves superior diagnostic outcomes compared to other methods, with an accuracy of 76.08%, *F*_H_ of 86.72%, and *F*_LCA_ of 85.39% in diagnosing 16 diseases and an accuracy of 82.07% in 5 coarse diseases.

In traditional diagnosis, after collecting the required patient indicators, it may take days for doctors to give the results. In contrast, intelligent diagnosis takes minutes or even less, for it can swiftly extract helpful information from a large amount of data. On the other hand, doctors have a one-third misdiagnosis rate [7], while our method has a higher accuracy rate.

However, based on experimental data, our method inevitably experiences misclassification. In practical application scenarios, if a physician cannot confirm the cause of a patient’s illness, our method could provide a possible direction to support the current treatment. Subsequently, the physician could reconfirm whether the model diagnosed the correct etiology according to the patient’s condition progression. If the model is misdiagnosed, the confirmed and corrected case is added to the dataset to train the model further and improve the prediction performance. In addition to FUO, our method can be used for other diseases where the data type is tabular, such as heart disease, breast cancer [22], and so on.

### Limitations

Due to the insufficient amount of data, our method has some limitations. As shown in Figure 4, diagnosing many diseases could be better. For instance, labels 5, 10, 11, 12, 14, and 15 (cytomegalovirus infection, rickettsia infection, anca-associated vasculitis, adult-onset Still disease, systemic inflammatory response syndrome, and hemophagocytic syndrome, respectively) all have an accuracy of less than 70%. Labels 5 and 11, in particular, have a correct diagnosis rate of only 50%, while label 12 has an accuracy of 45%. In addition, compared up to 200 FUO etiologies, the proposed method is only to be tested on 16 classes. The scarcity of many case data [54,55] makes it difficult to be verified on more other classes.

### Conclusions

This paper presents a diagnostic method for FUO using multipath feature selection and hierarchical classification. First, a hierarchical structure is constructed to identify the causes of FUO. A classification method is proposed to address the issue of interlevel error propagation in hierarchical classification, involving the preselection of multiple paths based on hierarchical prediction. Additionally, the L_1,2_ regularization constraint is used at each level within the hierarchical structure to facilitate feature selection. The objective is to eliminate redundant and interfering features, enhancing the method’s overall performance. Experimental findings indicate that the implementation of a hierarchical classification model significantly improves the accuracy of predicting FUO. Moreover, incorporating multiple path selection and feature selection further amplifies the effectiveness of the hierarchical classification model, offering a potential direction for the intelligent diagnosis of FUO.

Regarding future work, 2 aspects are considered. First, the FUO dataset should be expanded to improve prediction performance. Second, more optimal small-sample detection methods should be designed to increase the identification of rare diseases.

## Supplementary material

10.2196/58423 Multimedia Appendix 1 Detailed formula derivation of the proposed algorithm.

10.2196/58423 Multimedia Appendix 2 Detailed formulae for the derivation of assessment indicators.

10.2196/58423 Multimedia Appendix 3 Data used in this study.

## Abbreviations

ELM: extreme learning machine
FUO: fever of unknown origin
HNBP: hierarchical classification based on optimal N-paths
LR: logistic regression
ML: machine learning
RF: random forest
SVM: support vector machine
TIE: tree-induced error
