# Supplementary material for: A Pathological Diagnosis Method for Fever of Unknown Origin Based on Multipath Hierarchical Classification: Model Design and Validation
Source: JMIR Form Res. 2024 Dec 9;8:e58423. doi: 10.2196/58423 (PMC11649203; doi:10.2196/58423)
Supplement: Multimedia Appendix 1 [file formative-v8-e58423-s001.docx]

**The derivation of the L_1,2_ hierarchical feature selection**

Applying the sparse representation of feature selection to a tree hierarchy, feature selection can be performed based on whether the weight matrix of the features is zero or not.

Let the weight matrix ***ω****_i_* of each layer be divided into the sum of two components: ***W****_i_* and ***D****_i_*. Then the three parameters are substituted into the feature selection model of the sparse representation to obtain the minimization loss function of the ith layer concerning ***W****_i_* and ***D****_i_*:

$$\begin{matrix} J\left(W_{i},D_{i}\right)=\sum_{j=1}^{m_{i}} \frac{1}{n_{i,j}}\left\|X_{i,j}\omega_{i,j}-y_{i,j}\right\|^{2}+\lambda_{1}\left\|W_{i}\right\|_{1,2}+\lambda_{2}\left\|D_{i}^{T}\right\|_{1,2} \\ s.t\text{ }\omega_{i}=W_{i}+D_{i} \end{matrix}$$

where ***X****_i,j_* is the sample matrix of the ith layer belonging to the jth class, $\omega_{i,j}$ is the corresponding weight vector of ***X****_i,j_*, $n_{i,j}$ is the number of samples in ***X****_i,j_*, $y_{i,j}$ is the label of the class corresponding to $X_{i,j}$, is the L_1,2_ paradigm and λ_1_ and λ_2_ are non-negative parameters controlling the regularization. More specifically, ***ω****_i_*, ***W****_i_* and ***D****_i_* are:

where $i=1,2,\cdots,l$, *l* represents the number of levels in the tree hierarchy, $j=1,2,\cdots,m_{i}$, *m_i_* represents the number of classes in the ith layer, and *n* represents the sample feature dimension.

Traditional hierarchical feature selection considers different nodes as independent. It selects completely different subsets of features, whereas, in this paper, we propose selecting a portion of the same feature for every layer, known as shared features identified by ***W****_i_*, for different nodes in the same layer. Additionally, we select exclusive features for each node that are suitable for classification identified by ***D****_i_*. Figure S1 illustrates the process of choosing shared and exclusive features based on the dataset at ith layer in the hierarchy.

**Figure S1.** Feature selection process at the ith layer.


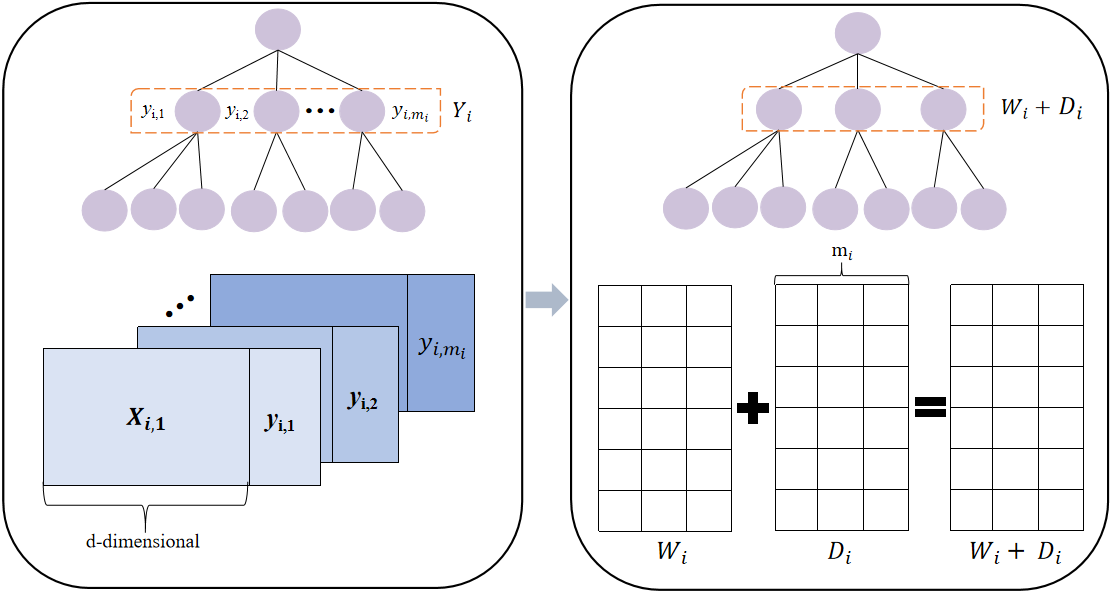


The procedure for solving the minimized loss function (1) is as follows: decompose equation (1) into *h*(***W****_i_*, ***D****_i_*) and *r*(***W****_i_*, ***D****_i_*):

$$\begin{matrix} h\left(W_{i},D_{i}\right)=\sum_{j=1}^{m_{i}} \frac{1}{n_{i,j}}\left\|X_{i,j}\left(W_{i,j}+D_{i,j}\right)-y_{i,j}\right\|^{2}, \\ \text{ }r\left(W_{i},D_{i}\right)=\lambda_{1}\left\|W_{i}\right\|_{1,2}+\lambda_{2}\left\|D_{i}^{T}\right\|_{1,2} \end{matrix}$$

where *h*(***W****_i_*, ***D****_i_*) is the empirical loss function, and *r*(***W****_i_*, ***D****_i_*) is the regularization term. That is, the objective minimization function (1) can be expressed as the differential term *h*(***W****_i_*, ***D****_i_*) with non-differential *r*(***W****_i_*, ***D****_i_*).

Taylor's formula can be approximated by a function *f*(*x*) with an nth-order derivative at $x=x_{0}$ using an nth-order polynomial in $(x=x_{0})$. Thus, let $H_{R,S,\eta}\left(W_{i},D_{i}\right)$ be the first-order Taylor expansion of *h*(***W****_i_*, ***D****_i_*) at (***R****_i_*, ***S****_i_*). By considering the sum of the squares of the Euclidean distances between $\left(W_{i},D_{i}\right)$ and $\left(R_{i},S_{i}\right)$ as the regularization term, $H_{R,S,\eta}\left(W_{i},D_{i}\right)$ can be expressed as:

$$\begin{matrix} H_{R,S,\eta}\left(W_{i},D_{i}\right)=h\left(R_{i},S_{i}\right)+\left\langle\frac{\partial h\left(R_{i},S_{i}\right)}{\partial R_{i}},W_{i}-R_{i}\right\rangle+\frac{\eta}{2}\left\|W_{i}-R_{i}\right\|_{F}^{2} \\ +\left\langle\frac{\partial h\left(R_{i},S_{i}\right)}{\partial S_{i}},D_{i}-S_{i}\right\rangle+\frac{\eta}{2}\left\|D_{i}-S_{i}\right\|_{F}^{2} \end{matrix}$$

The minimization loss function $J\left(W_{i},D_{i}\right)$ is solved iteratively using accelerated gradient descent until it converges. The solution $\left(W_{i}^{(k)},D_{i}^{(k)}\right)$ obtained by the following approximation operator under k iterations is computed as the optimal solution $W_{i}^{(*)},D_{i}^{(*)}$ through continuous iteration:

$$\left(W_{i}^{(k)},D_{i}^{(k)}\right)=\mathop{\arg\text{ min}}\limits_{W_{i},D_{i}} \text{H}_{W_{i}^{(k)},D_{i}^{(k)},\eta^{(k)}}(W_{i},D_{i})+r(W_{i},D_{i})$$

The iterative computational process can be expressed as follows:

$$\left\{\begin{matrix} R_{i}^{(1)}=W_{i}^{(0)},S_{i}^{(1)}=D_{i}^{(0)},t_{0}=1 \\ R_{i}^{(k+1)}=W_{i}^{(k)}+\alpha_{k}(W_{i}^{(k)}-W_{i}^{(k-1)}) \\ S_{i}^{(k+1)}=D_{i}^{(k)}+\alpha_{k}(D_{i}^{(k)}-D_{i}^{(k-1)}) \\ t_{k}=(1+\sqrt{t_{k-1}^{2}+1}/2) \\ \alpha_{k}=(t_{k-1}-1)/t_{k} \\ \eta_{k}=2^{m_{k}}\eta_{k-1}\text{ s.t }h(W_{i}^{(k)},D_{i}^{(k)})\leq H_{W_{i}^{(k)},D_{i}^{(k)},\eta^{(k)}}(W_{i}^{(k)},D_{i}^{(k)}) \end{matrix}\right.\text{, }k\geq1$$

where the last equation aims to determine the smallest non-negative integer, denoted as $m_{k}$, that satisfies the condition $h(W_{i}^{(k)},D_{i}^{(k)})\leq H_{W_{i}^{(k)},D_{i}^{(k)},\eta^{(k)}}(W_{i}^{(k)},D_{i}^{(k)})$.

Due to the decomposability of Equation (8), it can be transformed into two separate problems of proximal operators:

$$W_{i}^{(k)}=\mathop{\arg\text{ min}}\limits_{W_{i}} \frac{1}{2}\left\|W_{i}-\left(R_{i}^{(k)}-\frac{1}{\eta_{k}}\nabla_{R_{i}}h(R_{i}^{(k)},S_{i}^{(k)})\right)\right\|_{F}^{2}+\frac{\lambda_{1}}{\eta_{k}}\left\|W_{i}\right\|_{1,2}$$

$$D_{i}^{(k)}=\mathop{\arg\text{ min}}\limits_{D_{i}} \frac{1}{2}\left\|D_{i}-\left(S_{i}^{(k)}-\frac{1}{\eta_{k}}\nabla_{S_{i}}h(R_{i}^{(k)},S_{i}^{(k)})\right)\right\|_{F}^{2}+\frac{\lambda_{2}}{\eta_{k}}\left\|D_{i}^{T}\right\|_{1,2}$$

where $\nabla_{R_{i}}h(R_{i}^{(k)},S_{i}^{(k)})$ and $\nabla_{S_{i}}h(R_{i}^{(k)},S_{i}^{(k)})$ are the partial derivatives of $h(R_{i},S_{i})$ with respect to $R_{i}$ and $S_{i}$ at the point of$h(R_{i}^{(k)},S_{i}^{(k)})$.

The detailed proof of the convergence analysis of the method used to demonstrate this hierarchical feature selection is similar to that of the reference [34], which shows that the time complexity is $O(\frac{1}{k^{2}})$. That is, for any k ≥ 1, it can be obtained:

$$J(W_{i}^{(k)},D_{i}^{(k)})-J(W_{i}^{(*)},D_{i}^{(*)})=O(\frac{1}{k^{2}})$$

where $J(\cdot,\cdot)$ is the objective function and $J(W_{i}^{(*)},D_{i}^{(*)})$ denotes the optimal solution of Equation (3).

Ultimately, according to $W_{i}^{(*)}$ and $D_{i}^{(*)}$ we get the weight matrix $\omega_{i}^{(*)}$. The top N features are selected based on ordering the values of the elements in $\omega_{i}^{(*)}$.

Based on the above description and definitions, Algorithm 1 describes the process of hierarchical feature selection at each level based on L_1,2_ paradigm regularization.

| Algorithm 1：Hierarchical feature selection based on L_1,2_ paradigm regularization | | |
| --- | --- | --- |
| Input: sample matrix ***X***, hierarchical tree of classes  Output: the top N features of each layer in the hierarchy | | |
| for i=1 to l-1 do | | |
|  | $t_{0}=1,k=1$ | |
|  | while *k*<max do | |
|  |  | $t_{k}=(1+\sqrt{t_{k-1}^{2}+1}/2)$ |
|  |  | update $W_{i}^{(*)}$ and $D_{i}^{(*)}$ by Equation (10) and Equation (11)  *k*=*k*+1 |
|  | end  produce the optimal solution $W_{i}^{(*)}$ and $D_{i}^{(*)}$ after k iterations, and then get $\omega_{i}^{(*)}$ sort the values of each element in $\omega_{i}^{(*)}$to obtain a feature ordering for each class in this layer | |
| end  return: Feature ordering for each layer | | |

#### The derivation of multi-path hierarchical classification

Assume that the sample matrix of the dataset is represented by $X\in R^{m\times d}$, where *m* is the number of samples in the dataset and *d* is the selected data dimensions. Additionally, let $Y_{i}=\{y_{i,1},y_{i,2},...,y_{i.m_{i}}\}$ denote the set of classes in the ith granularity layer, where *m_i_* represents the number of granularity classes in that layer. The k pre-selected multipaths in this paper are limited to $1\leq k\leq\min(m_{i})$.

In the multi-path hierarchical classification process, the logistic regression is selected for the classifier, for it has many advantages: it is simple to implement, parallelizable, computationally inexpensive, fast, and can directly model the likelihood of classification. In addition, it not only predicts the classes but also provides the predicted probabilities for different classes. The likelihood for the jth class in a given ith classification subtask is:

$$p_{i,j}=\frac{1}{1+e^{-\omega_{j}^{T}x-b_{j}}}$$

where the *ω_j_* represents the weight of the feature in the sample data, and *b_j_* represents the bias. The weight (*ω_j_*) and the bias (*b_j_*) of the classification sub-tasks in each layer can be obtained by training on the training set. Usually, in tree hierarchies, the root node of the first layer generates all the nodes of the second layer. Therefore, the probability of the class nodes in the second layer can be calculated directly from the equation mentioned above.

First of all, considering the class inclusion relationship present in the upper and lower layers of the tree hierarchy, the probability of each subclass in each layer of the hierarchy is calculated sequentially. According to the logistic regression model and the weights and biases obtained from the training, the formula for the probability value of the hth subclass $c_{j,h}$ generated by the jth class on the (i-1)th layer at the ith layer is obtained as:

$$q(x)_{i,c_{j,h}}=\frac{1}{1+e^{-\omega_{c_{j,h}}^{T}x-b_{cj,h}}},c_{j,h}\in C_{j}$$

where $C_{j}$ is the set of all subclasses of the jth class.

Secondly, $P_{i,C_{j}}$ is utilized as the foundation for class ranking. In each layer, the top k pre-selected classes $Y_{i,k}$ are chosen based on their order until the top k pre-selected classes in the leaf node layer are acquired. The specific process is as follows: assuming that k preselected classes are selected from the previous (i-1)th layer, the subclasses generated by these k preselected classes in the ith layer can be obtained based on the relationship between the upper and lower layers. Subsequently, all the $k^{2}$ classes that are candidates to become preselected classes at the current ith layer can be obtained by the following equation (10):

$$\begin{matrix} P_{i,k_{C_{1}}}\cup P_{i,k_{C_{2}}}\cup\cdots P_{i,k_{C_{k-1}}}\cup P_{i,k_{C_{k}}}\Rightarrow P_{i,k^{2}} \\ \begin{matrix} Y_{i,k_{C_{1}}}\cup Y_{i,k_{C_{2}}}\cup\cdots Y_{i,k_{C_{k-1}}}\cup Y_{i,k_{C_{k}}}\Rightarrow Y_{i,k^{2}} \\ P_{i,k_{C_{i}}}=\max_{k} (P_{i,C_{i}}) \\ Y_{i,k_{C_{i}}}=\max_{k} (Y_{i,C_{i}}) \end{matrix} \end{matrix}$$

where $P_{i,k_{Cj}}$ represent the set of probabilities of the k preselected subclasses generated by the subclasses of class j in the (i-1)th layer at the ith layer. Similarly, $P_{i,k^{2}}$ represent the probabilities of the $k^{2}$preselected subclasses on the ith layer. $Y_{i,k_{C_{j}}}$ represent the set of the k preselected subclasses generated by the subclasses of class j in the (i-1)th layer at the ith layer, while $Y_{i,k^{2}}$ denotes the $k^{2}$ preselected subclasses on the ith layer.

The classes in the current ith layer are ranked based on the probability of occurrence among the *k*^2^ possible classes. The top k classes are then selected to form the pre-selected classes *Y_i,k_* for the ith granularity layer. This process can be described as:

$$P_{i,k}=\max_{k} \left(P_{i,k^{2}}\right)$$

Sequentially, starting from the top and moving downwards, the k pre-selected classes at the (i-1)th granularity layer result in *k*^2^ possible classes at the ith granularity layer. These are followed by the top k pre-selected classes with the highest probability values. Lastly, the k pre-selected classes are chosen at the leaf node layer.

Figure S2 details the multipath selection process at each layer(*k*=3). The three pre-selected classes chosen from the (i-1)th layer generate a set of three subclasses, *C*_1_, *C*_2_, *C*_3_ at the ith layer. A set of nine candidate pre-selected classes is obtained by selecting the first three classes according to their probability values. The selection of the three pre-selected classes in each layer is determined by the probability values assigned to the nine classes. This approach enables a multi-path hierarchical classification process, which proceeds from the top to the bottom layers. Ultimately, the three pre-selected classes in the leaf layer are identified.

**Figure S2.** Process of pre-selection *k*=3 pre-selection classes in hierarchical classification.


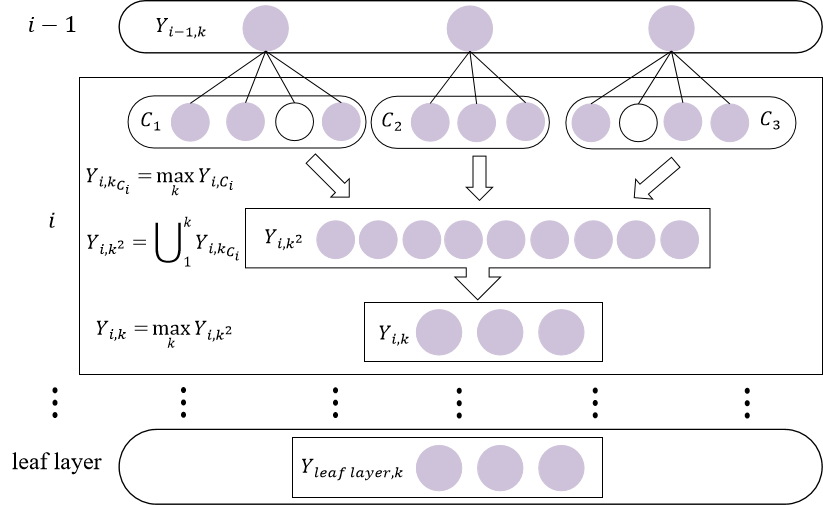


Finally, a classifier forecasts the ultimate classification within the k pre-selected classes derived from the multipath results. The optimal outcome is achieved when the true class of the predicted samples falls within one of the k pre-selected classes, and the base classifiers accurately classify among the k minority classes. Algorithm 2 outlines the process of multi-path hierarchical classification.

| Algorithm 2: Multi-path hierarchical classification | | | | |
| --- | --- | --- | --- | --- |
| Input: sample feature subset  Output: predict class  for i=1 to l do | | | |
| train the feature weights and biases of each class for each subclassification task at each layer using logistic regression in the test set | |
| end | | | | |
| for i=1 to l do | | | | |
| from the probability values of the preceding layer, the individual class probability values of the ith layer can be obtained using Eq. (14) | | |
| for t=1:*k* | | |
|  | get $P_{i,k^{2}}$ and $Y_{i,k^{2}}$ by Eq. (15) | |
|  | select *P_i,k_* and *Y_i,k_* of the ith layer by Eq. (16) | |
| end | | |
| end  determination of final prediction classes among pre-selected classes using base classifiers  return: predict class | | | | |
