# Supplementary material for: A Pathological Diagnosis Method for Fever of Unknown Origin Based on Multipath Hierarchical Classification: Model Design and Validation
Source: JMIR Form Res. 2024 Dec 9;8:e58423. doi: 10.2196/58423 (PMC11649203; doi:10.2196/58423)
Supplement: Multimedia Appendix 2 [file formative-v8-e58423-s002.docx]

(1) Hierarchical F_1_-measure (*F*_H_): Let Y denote the true label of sample X, Ŷ represent the predicted label of X, and $anc\left(Y\right)$ denote the set of parent nodes that accurately predicted samples. The hierarchical accuracy $P_{H}$, hierarchical recall $R_{H}$ and $F_{H}$ applicable to hierarchical structures are defined as follows:

$$\begin{matrix} P_{H}=\frac{\left|\hat{Y}_{aug}\cap Y_{aug}\right|}{\left|\hat{Y}_{aug}\right|} \\ R_{H}=\frac{\left|\hat{Y}_{aug}\cap Y_{aug}\right|}{\left|Y_{aug}\right|} \\ F_{H}=\frac{2\cdot P_{H}\cdot R_{H}}{P_{H}+R_{H}} \end{matrix}$$

where $Y_{aug}=Y\cup\text{anc}\left(Y\right)$, $\widehat{Y}_{aug}=\widehat{Y}\cup\text{anc}\left(\widehat{Y}\right)$, represented as the number of sets.

(2) *F*_H_ based on the closest ancestor (*F*_LCA_): The Lowest Common Ancestor (LCA) in the context of a tree-based hierarchical structure refers to the node that is the deepest and furthest from the root node among the common ancestors of the true class node and the predicted class node. Let $C_{aug}$ represent the set of nodes that lie along the path from the true class to the LCA node in the tree structure, and let $\hat{C}_{aug}$ represent the set of nodes along the path from the predicted class to the LCA node. The accuracy $P_{H}$($P_{LCA}$) and recall $R_{H}$($R_{LCA}$) metrics based on LCA are defined as follows:

$$\begin{matrix} P_{LCA}=\frac{\left|\hat{C}_{aug}\cap C_{aug}\right|}{\left|\hat{C}_{aug}\right|} \\ R_{LCA}=\frac{\left|\hat{C}_{aug}\cap C_{aug}\right|}{\left|C_{aug}\right|} \\ F_{LCA}=\frac{2\cdot P_{LCA}\cdot R_{LCA}}{P_{LCA}+R_{LCA}} \end{matrix}$$

$F_{H}$ metric counts multiple common ancestors, including the root node, which makes it difficult to accurately distinguish between different degrees of error at the lower level. On the other hand, $F_{LCA}$ metric only considers subtrees rooted in the actual node class and the LCA of the predicted node. This allows for a more precise comparison of the differences in splitting errors among the lower nodes. The larger $F_{LCA}$ value indicates that the fewer paths passed from the actual class nodes to the predicted class nodes, the lower the degree of errors and the better the classification results.

(3) Tree Induced Error (TIE): In classifying a hierarchical structure, varying prediction errors lead to distinct penalties. The TIE is a measure of the distance between the predicted class and the true class of a tree and is defined as follows:

$$TIE(Y,\hat{Y})=\left|E_{h}(Y,\hat{Y})\right|$$

where $E_{h}(Y,\hat{Y})$ represents the set of edges traversed from the true class node *Y* to the predicted class node $\widehat{Y}$ within a tree-like hierarchical structure, denotes the cardinality of this set, which refers to the number of edges.

(4) Accuracy:

$$accuracy=\frac{R_{N}}{N}$$

where $R_{N}$ represents the count of correct predictions, while *N* represents the total number of samples in the test set.

(5) Runtime (*T*)

Runtime encompasses both the duration of the training phase and the testing phase.
